# Clinical Epidemiology of Trauma Patients: A Retrospective Analysis of 3705 Consecutive Patients Treated at a Level I Trauma Center

**DOI:** 10.7759/cureus.80657

**Published:** 2025-03-16

**Authors:** Amit K Singh, Prabhaker Mishra

**Affiliations:** 1 Trauma, Sanjay Gandhi Postgraduate Institute of Medical Sciences, Lucknow, IND; 2 Biostatistics & Health Informatics, Sanjay Gandhi Postgraduate Institute of Medical Sciences, Lucknow, IND

**Keywords:** advanced trauma life support, golden hour, road traffic injuries, seatbelt and helmet regulations, trauma, trauma registry utilization

## Abstract

Background: Trauma is a leading cause of morbidity and mortality globally, particularly in low- and middle-income (LMIC) countries like India, where road traffic injuries (RTIs) dominate. Despite advancements in medical technology, trauma care remains underdeveloped due to resource limitations, inadequate pre-hospital care, and poor compliance with safety measures. This study aimed to analyze the clinical-epidemiological profile of trauma patients and develop strategies for effective trauma prevention and management.

Methods: A retrospective review of 3,705 trauma patients admitted to a level I trauma center between July 2018 and June 2024 was conducted. Data on demographics, injury mechanisms, triage priority, treatment outcomes, and resource utilization were analyzed. Patients were managed following Advanced Trauma Life Support (ATLS) protocols, with multidisciplinary care and trauma registry utilization.

Results: RTIs accounted for 67.3% of cases, with two-wheeler accidents being the most common (84.7%). Males comprised 78.3% of patients, with a mean age of 37.5 years. Alcohol intoxication was present in 41.9% of cases. Head injuries (1663/3705; 44.9%) and polytrauma (719/3705; 19.5%) were prevalent, with a mortality rate of 4.0%. ICU admission was required for 58.4% (n=2165) of patients, and of these, 992 (45.8%) needed mechanical ventilation. Of the 2,111 two-wheeler-related accidents, only 33% (696) wore helmets and of the 201 four-wheeler accidents, seatbelts were worn by only 41% (n=83). Low compliance with helmet and seatbelt use exacerbated injury severity.

Conclusion: The study emphasizes the critical need for tighter enforcement of seatbelt and helmet regulations, improved pre-hospital care systems, and improved road safety measures. To lower trauma-related morbidity and mortality in India, it is essential to build trauma registries, strengthen the infrastructure for trauma care, and put evidence-based policies into place.

## Introduction

Trauma ranks as the third most prevalent cause of death worldwide for all age groups and is a major source of morbidity and mortality, especially for those under 40 years of age [1]. Trauma is becoming more widely acknowledged by health professionals as a contemporary epidemic, particularly in low- and middle-income nations. For example, although possessing just 1% of the world's motor vehicles, India is responsible for 10% of all road traffic accidents worldwide, with over 4.5 lakh accidents and 1.5 lakh fatalities per year [2]. Rapid urbanization, industrialization, and motorization are the main causes of this unequal load. In addition to ruining affected families, trauma has become a silent killer that puts a tremendous burden on the nation's healthcare system. Compared to the United States, where healthcare spending accounts for 16.57% of GDP, India's healthcare spending in 2021-2022 was only 3.28% [3]. In India, trauma care is still lacking despite major advances in medical technology such as imaging and medical equipment. The problem is made worse by issues like scarce resources, a heavy clinical load, and patient financial limitations [4]. 

Effective trauma management is also severely hampered by India's geographically dispersed population, the bulk of whom live in rural areas. Poor pre-hospital treatment is frequently the result of inadequate rural healthcare infrastructure, which raises the morbidity and fatality rates associated with trauma. 

The pre-hospital period, particularly the first hour after injury, often referred to as the “golden hour,” is critical, as most trauma deaths occur during this time [5]. Given the high prevalence of trauma and the severe consequences of delayed care, preventive measures are essential. Prior research has demonstrated that a mix of environmental, socioeconomic, and demographic factors affects the prevalence of trauma [6-8]. Enhancing the standard of trauma care and creating successful preventative plans require a methodical examination of these variables [9,10]. In view of these difficulties, the current study intends to ascertain the clinical-epidemiological characteristics of trauma survivors, examine injury trends, and develop a thorough plan for efficient trauma management and prevention in a regional trauma center.

## Materials and methods

We reviewed retrospective trauma registry data from level I apex Trauma Center of Sanjay Gandhi Postgraduate Institute of Medical Science, Lucknow, India, to identify all patients (adults and children) admitted with trauma between July 2018 and June 2024. The Sanjay Gandhi Postgraduate Institute of Medical Science Ethics Committee approved the study protocol (approval number: PGI/BE/912/2024). The study adheres to the Strengthening the Reporting of Observational Studies in Epidemiology (STROBE) guidelines for reporting observational studies.

Inclusion and exclusion criteria

Inclusion criteria included all trauma patients admitted to trauma emergency. Exclusion criteria included patients who were brought dead, patients with incomplete case records, or lost to follow-up. As burns, hangings, drownings, and animal bites are not treated in our center, these patients were also not considered for the study.

Data collection

Case records were examined to determine demographic information, including age, gender, mechanism of injury, hemodynamic status, transfusion requirements, computed tomography (CT) scan results, time between initial diagnosis and surgery, incident time, triage priority level, length of hospital and Intensive care unit (ICU) stay, number of days on a ventilator, complications, and final outcome.

Trauma patient management process

Trauma patients were triaged according to the Advance Trauma Life Support (ATLS) protocol in the trauma emergency department. Triage priority I trauma patients were those who had immediate life-threatening injuries and required immediate intervention. These patients were assigned to the red zone and treated accordingly. Triage priority II trauma patients were those who had no airway, respiratory, or circulatory compromise; they were assigned to the yellow zone. Triage priority III patients were those who had minor injuries and did not require admission.

After the initial examination, relevant radiological examinations, such as extended focussed assessment with sonography in trauma (E-FAST), chest X-ray, pelvic X-ray, CT of the head and pelvis, and all basic blood tests (complete blood count, renal function test, liver function test, coagulation profile, serum electrolytes, arterial blood gas), were performed to assess injuries.

Patients with hemodynamic instability were first resuscitated in the emergency room. For resuscitation, 1 liter of Ringer's lactate fluid was administered first, followed by a transfusion of cross-matched packed red blood cells and fresh frozen plasma in a 1:1 ratio. To maintain a mean arterial pressure of > 70 mmHg, an infusion of norepinephrine was started and titrated accordingly. Patients having a Glasgow coma scale (GCS) <8 were electively intubated.

Information about the center

Our center has 24 x 7 dedicated services of neurosurgeons, orthopaedic surgeons, trauma surgeons, and oral and maxillofacial surgeons (OMFS). Infrastructure includes a 24-bed ICU, five modular operating theatres, 128-slide CT scan. Initial assessment and resuscitation are performed in trauma emergency by the emergency department (ED) team and after 24 hours, the patient is shifted either to the respective specialty ward or ICU depending on the hemodynamic condition and injury. For the management of polytrauma patients, multispecialty trauma teams consisting of one faculty each from neurosurgery, orthopaedics, trauma surgery, OMFS, anaesthesia, radiology, and plastic surgery have been constituted. Trauma review meetings are held every day and a multidisciplinary team evaluates patients and plans management as per the ATLS protocol.

Statistical analysis

Descriptive statistics of the continuous variables are presented as mean (standard deviation (SD)), whereas categorical variables are presented as frequency (percentage). The chi-square test was used to compare the proportions between the two groups. A bar diagram and pie chart were used to present the results as appropriate. P value<0.05 was considered statistically significant. Statistical analysis was performed using IBM SPSS Statistics for Windows, Version 23.0 (Released 2015; IBM Corp., Armonk, New York, United States).

## Results

During the study period of six years (July 2018 - June 2024), our trauma emergency department admitted 3,731 different kinds of trauma patients. Nineteen patients were brought in dead and were not included in the study. Records of seven patients were found to be incomplete; they were also excluded. Therefore, the final cohort comprised 3,705 patients. Among these, there were 459 (12.3%) patients who were seen in the non-acute area and discharged after initial assessment. All age groups were captured, ranging from two months to 102 years with a mean age of 37.5 years (SD 22.56). Pediatric trauma comprised 309 (8.3%) patients, while adult trauma comprised 3,396 (91.7%) patients. Male patients (n=2,901; 78.3%) outnumbered female (n=804; 21.7%) with an M: F ratio of 3.6:1, which indicated a significantly higher proportion of males (p<0.001) (Table 1)

**Table 1:** Demographics and trauma characteristics of the patients included in the study (N=3705) Data presented as frequency (percentage) except for age, which is presented as the mean

Category	Number	Percentage
Pediatric Trauma (<18 yrs)	309	8.3
Adult Trauma (≥18 yrs)	3,396	91.7
Sex		
Male	2,901	78.3
Female	804	21.7
Mean Age	37.5 years	—
Time of Trauma Occurrence Between 6 AM – 5 PM	2704	73
Patients Arriving Between 7 PM – 06 AM	2556	69
Alcohol Intoxication	1,553	41.9

Mostly, young patients reported to the trauma center. Seventy-three percent of trauma (n=2705) occurred between 6 a.m. and 5 p.m.; however, almost 2557 (69%) of patients presented to the ED between 7 p.m. and 6 a.m. Alcoholic intoxication was found in 1553 (41.9%) of patients. Most of these 1428 (92%) patients presented during the night hours after 11 p.m. 

Triage I category was assigned to 459 (12.4%) patients presenting with life-threatening injuries. The majority of patients were in Triage II categories (n=2,371; 64%), while 874 (23.6%) patients were categorized in Triage III, requiring treatment for minor injuries or admission only for short observation. There was a significantly higher proportion of patients who presented with road traffic injury (RTI) as compared to other types of injury (67.3% vs 32.7%, p<0.001) (Table 2)

**Table 2:** Injury patterns, causes, and clinical outcomes (N=3705)

Category	Number	Percentage
Triage Classification		
Triage I (Life-threatening)	459	12.4
Triage II (Moderate severity)	2,371	64.0
Triage III (Minor injuries)	874	23.6
Causes of Trauma		
Road Traffic Injuries (RTI)	2,493	67.3
- Two-Wheelers	2,111	84.7 (of RTIs)
- Four-Wheelers	201	8.1 (of RTIs)
- Pedestrians	154	6.2 (of RTIs)
Falls from Height	396	10.7
Falls in Bathrooms	255	6.9
Assault	351	9.5
Workplace Injuries	174	4.7
Animal Injuries	22	0.6
Sports Injuries	11	0.3
Medical Interventions		
Endotracheal Intubation	217	5.8
Chest Tube Drainage	329	8.8
Resuscitative Thoracotomy	4	—
Hospital Outcomes		
ICU Admissions	2,165	58.0
Mechanical Ventilation Required	992	45.8
Blood Transfusion Required	389	10.4
Overall Mortality in ICU	303	13.9
Deaths in Emergency Bay	149	4.0

The majority of trauma was caused by RTI (n=2,493; 67.3%), followed by falls from height (n=396; 10.7%), falls in the bathroom (n=255; 6.9%), assault (n=351; 9.5%), workplace injuries (n=174; 4.7%), animal injuries (n=22; 0.6%), and sports injuries (n=11; 0.3%). Out of these 2,493 (67.3%) RTI, two-wheelers accounted for 2,111 (84.7%) patients, four-wheelers for 201 (8.1%), pedestrians for 154 (6.2%), and others for 25 (1%). Only 696 (33%) patients wore helmets while riding two-wheelers, and the majority of them (n=640; 92%) were driving the vehicle. Seat belts were worn by 83 (41%) four-wheel drivers.

Of the 255 patients with falls in the bathroom, 195 (76.4%) were elderly people aged >60 years. Fractures of the neck of the femur, followed by intertrochanteric fractures, were the most common findings. 

**Figure 1:**
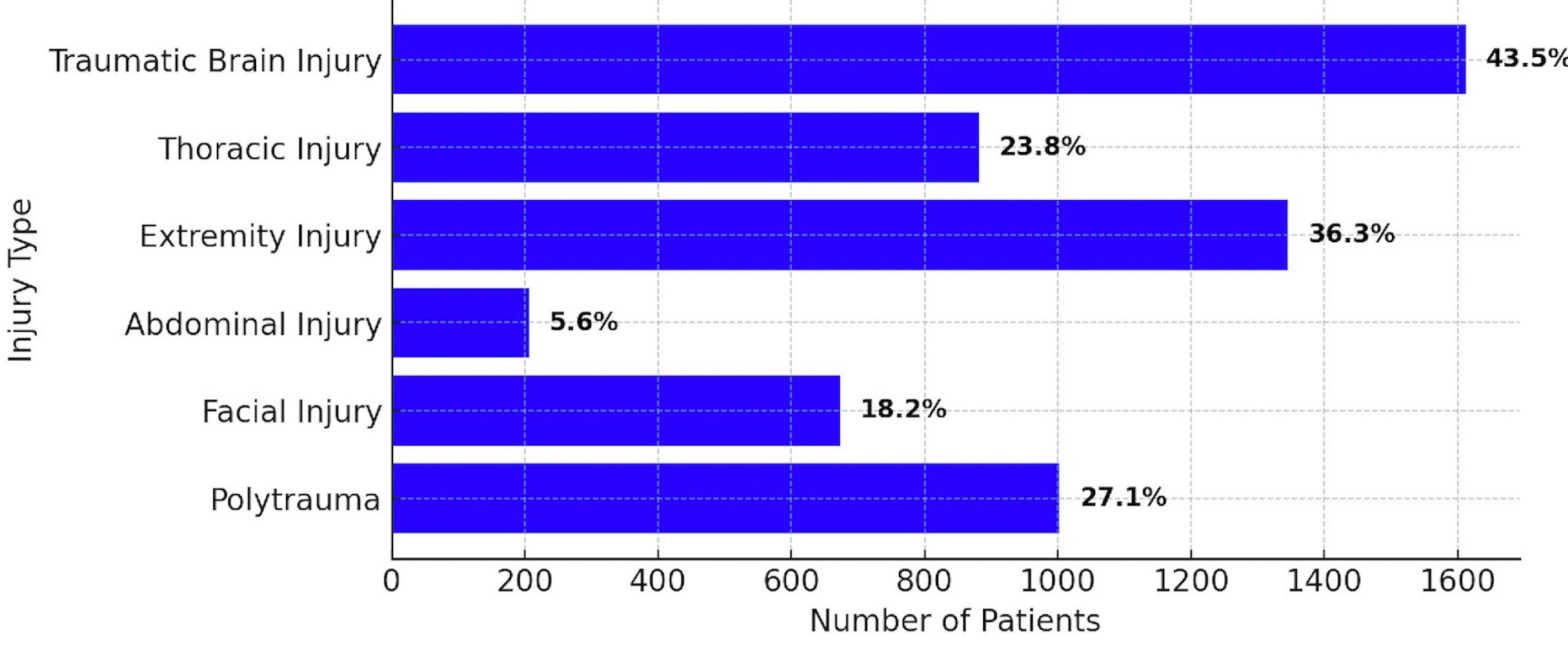
Injury pattern in referred trauma patients (multiple response)

Since ours is a tertiary level I trauma center, two-thirds (n=2471) of our patients were received from referral hospitals in a 500 km radius. The majority of referral patients had a traumatic brain injury (n=1073; 43.5%), followed by polytrauma (n=668; 27.1%), thoracic injury (n=587; 23.8%), and abdominal injury (n=143; 5.6%). During emergency resuscitation, endotracheal intubation was done in 217 (5.8%) patients. Intercostal tube drainage was performed in 329 (8.8%) patients, either for hemothorax or pneumothorax. Resuscitative thoracotomy was performed on four patients with open chest wounds. Figure 1 shows the pattern of injuries.

Traumatic brain injury and the majority of polytrauma cases were mostly seen in two-wheeler accidents, followed by falls from height. Of the total of 1663 head cases (including 1073 referral cases), 1119 (67.2%) involved two-wheelers, and 387 (23.2%) involved falls from a height. In a similar vein, of the 719 polytrauma cases (including 688 referral cases), 228 (31.7%) involved two-wheelers and 71 (9.9%) involved falls from a height. 

The trauma emergency team alone treated and discharged 675 (18.2%) patients, while the remaining 3,030 (81.2%) required further evaluation by surgical teams of different specialties (Table 3).

**Table 3:** Post-injury trauma outcome of the included patients The percentage calculations for Mechanical Ventilation, Blood Transfusion, Percutaneous Tracheostomy, Deaths in Emergency Bay, and ICU Mortality are based on the total number of patients who required further evaluation by specialists (n=3030)

Treatment Outcomes	Number	Percentage
Treated and discharged by the ED trauma team	675	18.2%
Required further evaluation by specialists	3030	81.8%
ICU Admissions	2165	58.4%
Mechanical Ventilation	992	45.8%
Blood Transfusion	389	10.4%
Percutaneous Tracheostomy	129	5.9%
Deaths in Emergency Bay	149	4.0%
ICU Mortality	303	13.9%

**Figure 2:**
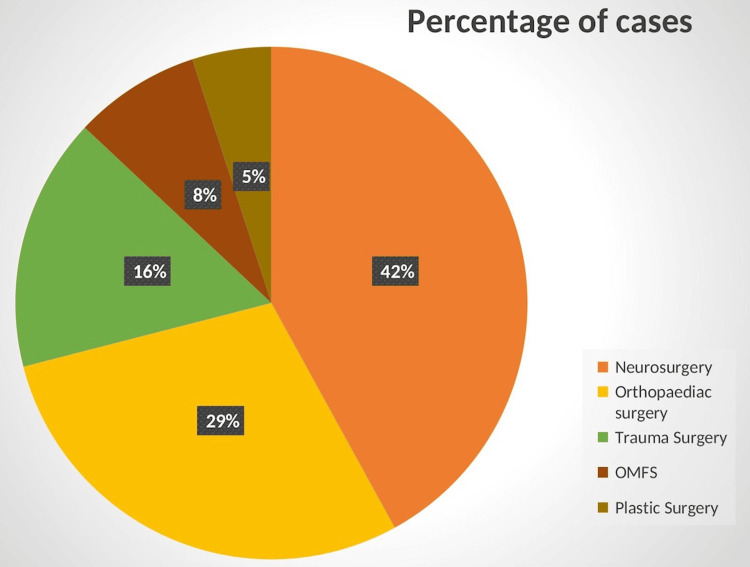
Distribution of various specialities in trauma management

Figure 2 shows the commonly involved specialties in the management of trauma patients.

**Figure 3:**
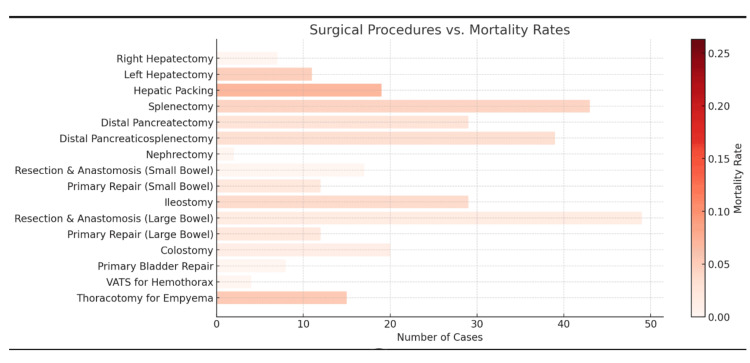
Visual chart showing distribution of mortality rates according to surgical procedures

Blood transfusion was required in 389 (10.4%) patients who presented with hypovolemic shock. Out of these 389, 63 (16.1%) were non-responders, 87 (22%) were transient responders, and 239 (61.4%) recovered from shock after receiving the transfusion. There were 149 (4.0%) deaths in the emergency bay. Of these 149 deaths, 63 (42.2%) died within 24 hours of admission, while 86 (57.8%) died after 24 hours of admission. The total number of patients admitted to the trauma ICU was 2,165 (58%). This unusually high number of admissions is because all electively operated patients of each specialty are monitored in the ICU for the initial 24 hours. During the study period, the number of major surgeries performed by the trauma specialty included 3,427 by orthopaedics, 1,539 by neurosurgery, 316 by trauma surgery, 259 by OMFS, and 47 by plastic surgery (Figure 3).

In the ICU, mechanical ventilation was required in 992 (45.8%) patients. Patients suffering from severe head injury, polytrauma, and thoracic trauma occupied the ventilator for the majority of the time. The mean ventilator days were 12.5 days (range 1-42 days), the mean ICU stay was 18.9 days (1-78 days), and the mean hospital stay was 26.2 days (1-120 days). The overall mortality in the ICU was 303 (13.9%). For prolonged ventilation, percutaneous tracheostomy was performed in 129 (5.9%) patients. To provide a higher level of nursing care, the patient-to-nurse ratio was 2:1. Criteria for trauma ICU admission include patients with hemodynamic instability, the requirement of mechanical ventilation, severe head injury, polytrauma with Injury Severity Score (ISS) > 15, the presence of coagulopathy, major metabolic derangement, and multi-organ failure.

## Discussion

This study is a descriptive cross-sectional analysis of all trauma patients managed in a level I trauma center. According to the WHO, motor vehicle crashes, falls, and interpersonal violence are among the top 20 worldwide causes of mortality and disability [11]. Over 90% of injury-related deaths occur in LMICs, where trauma disproportionately impacts communities [12]. 

RTIs have increased as a result of rapid motorization in India without a commensurate focus on safety. The population and economy of the nation have expanded quickly, and accordingly, the amount of motor vehicles has increased, especially two-wheelers. The count of registered two-wheelers rose from 47.5 million in 2003 to 263.3 million in 2022, whereas the number of four-wheelers grew from 8.5 million to 49. 1 million [13]. The National Crime Bureau reports that the number of road accidents rose from 422,659 in 2021 to 472,467 in 2022, and that the number of unintentional fatalities jumped from 173,860 to 194,347 in the same period [14]. In 2022, the number of unintentional deaths increased from 29.1 to 31.2 per 100,000 people. Critical concerns leading to the increase in road traffic accident deaths in India have been brought to light by reviews conducted by independent and government entities [15,16]. Notably, two-wheelers and pillion riders are involved in 30-40% of fatalities, primarily as a result of lax or non-existent helmet regulations [17]. There are notable differences between urban and rural regions; poor visibility, crossing facilities, and walking spaces are responsible for one-third of pedestrian fatalities. Speeding, drinking, lax traffic policing, and badly constructed or maintained roads are further contributing causes.

Additionally, the creation of efficient trauma treatment systems has been hampered by the absence of formalized emergency medical services (EMS), injury data, and research. Ineffective preventative tactics are the outcome of fragmented efforts by isolated entities, which worsen the issue. On the other hand, trauma registries, which are still in their infancy in India, have dramatically decreased trauma-related morbidity and mortality in nations like the United States [18]. Effective prevention and control measures require a complete trauma care system that includes pre-hospital, hospital, and post-hospital treatment. Studies including those by Mbanjumucyo et al. [19] and O'Reilly et al. [20] emphasize the importance of epidemiological data on trauma and injury for enhancing care.

The results of the current study highlight the significant prevalence of trauma in India, especially RTIs, with 67.3% of all trauma cases in the study being related to RTIs. This is consistent with worldwide patterns, which also show that RTIs constitute a major contributor to trauma-related morbidity and mortality, particularly in LMICs [21]. In the current study, the majority of RTIs (84.7%) involved two-wheeler accidents, underscoring the critical need for tougher enforcement of helmet regulations and other traffic safety precautions. The severity of injuries and death rates is exacerbated by the concerning low compliance rates for seatbelt (41%) and helmet (33%) use.

With a mean age of 37.5 years and a male preponderance of 78.3%, the demographic profile of trauma patients in the current study was in line with existing literature that indicates young males are disproportionately impacted by risk-taking behaviors, occupational risks, and increased exposure to traffic [22,23]. A study by Fararoei et al. in Iran also showed the age group of 24-44 years to be the most vulnerable population to suffer from RTIs [24].

Of the trauma patients in this study, 41.9% reported being intoxicated, especially at night, which highlights the need for public health initiatives that address alcohol use and drunk driving. With falls from height accounting for 10.7% of cases, particularly among the elderly, fall prevention measures like home safety improvements and public awareness campaigns are crucial. Also noteworthy were the high numbers of traumatic brain injuries and polytrauma cases, which call for multidisciplinary treatments and advanced trauma care facilities. 

The mortality rate in our study was 4.0%, and 42.2% of deaths occurred within 24 hours after admission. This underscores the importance of prompt pre-hospital care and efficient emergency resuscitation. One of the biggest obstacles to lowering trauma-related mortality in India is still the absence of a formalized emergency medical service. Results could be greatly improved by fortifying pre-hospital care systems, which include enhancing ambulance services and educating first responders. With 45.8% of patients requiring mechanical ventilation and 58.4% requiring ICU admission in the current study, trauma care is resource-intensive. Long hospital and ICU stays put additional strain on the healthcare system, especially in places with low resources. This emphasizes the necessity of funding trauma care infrastructure, which includes specialized trauma centers, skilled staff, and cutting-edge medical technology

As this study shows, creating a trauma registry is an essential first step in enhancing trauma care and preventive tactics. Trauma registries offer useful information on injury trends, outcomes, and resource use, which supports evidence-based policymaking and targeted treatments. For LMICs like India, the effectiveness of trauma registries in high-income nations like the United States serves as an example [25]. 

Even though the trauma landscape is dominated by RTIs, other injuries also account for a sizable percentage of trauma cases and should receive particular consideration. These injuries have distinct epidemiologic patterns, risk factors, and preventive measures. These include falls, assaults, workplace injuries, and animal injuries. Our study found falls from a height in 396 (10.07%) patients. These injuries frequently happen at work, such as on building sites, or while engaging in recreational activities. A total of 255 (6.9%) falls occurred in the restroom; hip fractures are frequently the consequence of bathroom falls, which mainly affect the elderly. Osteoarthritis and femoral neck fractures are also a concern. Common contributing factors include slick surfaces, inadequate lighting, and a dearth of assistance [26]. Strategies for prevention include the use of protective gear, enforcement of workplace safety regulations, and providing training to employees who are at risk for falls from heights. For bathroom falls, public awareness campaigns, community-based fall prevention programs for the elderly, and use of non-slip mats and grab bars in bathrooms.

Among young men in particular, assaults including intentional harm and interpersonal violence are a major cause of trauma [27]. These injuries frequently include soft tissue injuries, facial fractures, and head trauma. Major causes include urban violence, socioeconomic inequality, and alcohol intoxication. Prevention techniques include public health initiatives that encourage resolving disputes and lowering alcohol intake, interventions centered in the community to combat substance abuse and violence, enforcing tougher penalties for offenders, and bolstering law enforcement.

Workplace injuries are common in industrial and construction settings mainly due to the involvement of machinery, falls, or exposure to hazardous materials [28]. Frequent injuries include traumatic amputations, fractures, and crush injuries. Strict adherence to workplace safety regulations, frequent safety instruction for both employers and employees, and utilizing ergonomic tools and personal protective equipment (PPE) can minimize such injuries.

Limitations of the study

The study was performed in a single center in a specified area for a specific period which can be a reason for bias owing to the demography of people living in that particular area. The study was retrospective and observational in nature, which can be limitations because of potential for bias, reliance on existing data quality, and difficulty in controlling for confounding factors.

## Conclusions

The results of this study demonstrate the significant impact of trauma, particularly RTIs, and the urgent need for measures to improve road safety, trauma management systems, and pre-hospital care. Because brain injuries, polytrauma, and alcohol-related trauma are so common, it is important to take a multidisciplinary approach to trauma care and implement targeted prevention strategies. Implementing evidence-based policy and setting up trauma registries are necessary to lower trauma-related morbidity and mortality. To accomplish this goal, pre-hospital care must be strengthened and safety procedures must be followed. Despite being frequently disregarded in trauma discussions, non-RTIs also pose a serious public health concern. A comprehensive strategy combining prevention, public education, healthcare system strengthening, and policy advocacy is needed to address these injuries. Targeted strategies to lower assaults, falls, workplace injuries, and other non-RTIs can greatly lessen the overall burden of trauma.
